# Molecular insights of drug-resistant tuberculosis: genetic mutations and their profile

**DOI:** 10.3389/fmicb.2025.1669327

**Published:** 2025-10-03

**Authors:** Amresh Kumar Singh, Nandini Singh, Sushil Kumar, Ashwini Kumar Mishra, Narendra Pratap Singh

**Affiliations:** ^1^Department of Microbiology, Baba Raghav Das Medical College, Gorakhpur, Gorakhpur, India; ^2^Department of Zoology, Deen Dayal Upadhyaya Gorakhpur University, Gorakhpur, India; ^3^Department of Tuberculosis and Chest, Baba Raghav Das Medical College, Gorakhpur, Gorakhpur, India; ^4^Department of Microbiology, Baba Raghav Das Medical College, Gorakhpur, Gorakhpur, India

**Keywords:** drug-resistant tuberculosis, genetic mutation pattern, line probe assay, isoniazid resistance, rifampicin resistance, molecular diagnostics

## Abstract

**Introduction:**

Drug-resistant tuberculosis (DR-TB) poses a significant public health threat, with molecular diagnostics playing a pivotal role in understanding the genetic mechanisms of resistance. This study focuses on the patterns of genetic mutations observed in DR-TB cases, with the aim to identify key mutations associated with resistance to rifampicin (RIF) and isoniazid (INH).

**Methodology:**

A total of 6,954 non-duplicate clinical samples were obtained from individuals of all age groups, categorized as TB and DR-TB, from seven linked districts between June 2022 and May 2024. The samples were transported under cold chain conditions to an intermediate reference laboratory. TB was confirmed using fluorescence microscopy, and 1,998 sputum-positive samples were analyzed using line probe assay for characterization of genetic mutations.

**Results:**

Among the analyzed cases, a total of 136 cases of DR-TB were identified. This included 57 cases (41.92%) of multidrug-resistant TB (MDR-TB), 73 cases (53.68%) of INH monoresistance, and 6 cases (4.4%) of RIF monoresistance. The analysis revealed a high prevalence of *rpo*B MUT3 (S531L) mutations in 52 cases (82.25%), which is associated with RIF resistance. In high-level INH (*kat*G gene mutation) resistance noted in 83 (63.35%) cases, *kat*G MUT1 (S315T1) was predominant, while low-level INH resistance (*inh*A gene mutation), *inh*A MUT1 (C-15T) mutation, was found in 29 (22.13%) cases. Maharajganj and Deoria reported the highest prevalence of *rpo*B MUT3 (S531L) mutations, while Kushinagar and Sant Kabir Nagar exhibited higher rates of *kat*G MUT1 (S315T1) mutations. Other regions showed notable distribution of *rpo*B, *kat*G, and *inh*A gene mutations.

**Conclusion:**

The high prevalence of mutations such as *rpo*B MUT3 (S531L) and *kat*G MUT1 (S315T1) highlights the need for integrating molecular tools into routine workflows to identify genetic mutations. District-specific mutations emphasize the influence of local epidemiological factors on resistance patterns, necessitating region-specific interventions. Continuing research into regional resistance trends are vital to addressing the global DR-TB burden effectively.

## 1 Introduction

*Mycobacterium tuberculosis* complex (MTBC) causes tuberculosis (TB), which is a major public health concern (Singh et al., 2022b). The WHO reported that 10.8 million individuals globally were diagnosed with TB in 2023, accounting for 134 new cases per 100,000 people (WHO, 2024). In India, 2.55 million TB cases were reported in 2023 (India TB Report, 2024). Resistance in MTBC strains to anti-TB drugs causes drug-resistant TB (DR-TB), which is driving the ongoing TB epidemic and posing challenges to public health (Loddenkemper and Murray, 2021). Multidrug-resistant TB (MDR-TB) occurs when bacteria become resistant to primary TB medications, specifically rifampicin (RIF) and isoniazid (INH), while extensively drug-resistant tuberculosis (XDR-TB) develops when resistance extends to both first-line drugs and second-line treatments such as fluoroquinolones (FQ) and group A medications (Mase and Chorba, 2019). Resistance to INH can be further categorized as high level or low level resistance, based on the underlying genetic mechanism. Low-level INH resistance is linked to mutations in the *inh*A promoter region, specifically C-15T, while high-level INH resistance is caused by mutations in the *kat*G gene, specifically S315T (Brown and Schult, 2024). Inadequate anti-tubercular treatment (ATT) or improper dosing may contribute to the development of DR-TB. The annual India TB Report, 2024 indicates that DR-TB accounted for 2.5% of all TB cases (India TB Report, 2024).

India accounted for approximately 26% of global TB cases in 2023, substantially higher than the figures for China, Indonesia, or Pakistan. Additionally, India contributed to 27% of the global incidence of MDR/Rifampicin-resistant tuberculosis cases in 2023, followed by Indonesia, the Russian Federation, and China, highlighting its disproportionate share of the DR-TB burden (WHO, 2025). However, between 2015 and 2023, India reported a gradual decline in estimated TB incidence, i.e., up to 16% reduction (India TB Report, 2024). During the same period, the mortality rate of TB decreased by 18%, from 32 to 26 per 100,000 population. Despite this progress, India still accounted for 2.55 million reported TB cases in 2023, which is the highest burden globally. The number of MDR/RR-TB patients also increased sharply, from 28,096 in 2015 to over 63,000 in 2023, underscoring the persistent challenge of drug resistance (Report, Annual India TB, 2016).

Molecular methods such as line probe assays (LPAs) are the most effective tools for rapid detection of drug resistance. LPAs can guide clinicians in personalizing appropriate therapy for patients with DR-TB (Suzana et al., 2021). The development of LPAs marks a notable progress in TB diagnostics, allowing for more rapid and accurate identification of drug resistance when compared to conventional culture-based approaches. Geographical and demographic assessments of DR-TB are valuable for examining the spatial distribution of TB and deploying sophisticated diagnostic methods such as LPA. These assessments detect genetic changes in particular codons, which can modify protein configuration, possibly resulting in resistance against second-line TB medications. However, these tests require specialized laboratory infrastructure and may miss rare mutations outside target regions. Whole-genome sequencing (WGS) and next-generation sequencing (NGS) provide comprehensive insights into transmission dynamics and emerging mutations, although their high cost and technical expertise requirements restrict routine use in India.

RIF is one of the most significant anti-TB medications due to its extremely potent bactericidal action against MTBC. RIF targets the *rpo*B gene, which encodes the DNA-dependent RNA polymerase β-subunit (Taylor et al., 2023). The molecular mechanism of drug resistance consists of a conformational shift caused by mutated *rpo*B, which affects RIF’s binding affinity at the β-subunit of the RNA polymerase (RNAP) (Miotto et al., 2018). INH is also a primary anti-TB drug that exhibits two types of resistance: mutation in *kat*G gene (catalase-peroxidase enzyme), i.e., high-level resistance, and mutation in *inh*A gene (fatty acid synthesis), known as low-level resistance (Brown and Schult, 2024). In addition, mutations in genes such as *gyr*A and *gyr*B (DNA-gyrase) and *rrs* (16s rRNA) cause second-line drug resistance (Singh et al., 2024). Molecular and culture tests are specifically useful in the diagnosis of MDR-TB because they provide essential data for adjusting treatment.

MDR-TB or XDR-TB also exhibits distinct geographical patterns, and identifying the regional hotspots by geographic evaluation makes it possible to allocate resources more appropriately and customize medication regimens to local resistance profiles, both of which are essential for treatment effectiveness and transmission management.

In this study, we employed molecular diagnostics, specifically LPA, to identify mutational patterns, integrating clinical and demographic profiling to better understand regional epidemiology. Our study highlighted the burden and distribution of genetic mutational pattern in primary first-line drugs due to limited information on district-level mutation profiles of drug-resistant *M. tuberculosis* in Northern India, particularly under programmatic conditions. By combining molecular diagnostics with demographic and clinical profiling, we found that DR-TB is more prevalent in previously treated patients and linked to specific risk factors like workplace exposure. The study underscores the critical significance of precise identification of drug resistance in MTBC to increase treatment effectiveness. Our study also highlighted that early detection and monitoring of resistance pattern strengthen TB control programs in high-burden regions, as understanding these molecular patterns is crucial for guiding early diagnosis, individualized treatment, and effective public health interventions.

## 2 Methodology

### 2.1 Sample collection

Our study is a prospective cross-sectional study that was conducted in intermediate reference laboratory (IRL), Baba Raghav Das Medical College (BRDMC), Gorakhpur, Uttar Pradesh, India. We examined 6,954 suspected TB samples from May 2022 to June 2024, which were received via cold chain by postal service from seven districts (Gorakhpur, Kushinagar, Deoria, Maharajganj, Sant Kabirnagar, Siddharthnagar, and Basti). TB isolates were examined for First-Line Probe Line Assay after florescence microscopy. Patient data, including age, sex, district, and workplace, and prior TB and anti-tubercular treatment (ATT) history, were collected using a structured proforma and hospital information system (Singh et al., 2024).

### 2.2 Inclusion and exclusion criteria

Patients with bacteriologically confirmed TB and patients with strong clinical suspicion of TB referred under National Tuberculosis Elimination Programme (India), provided adequate clinical samples were available for testing, were included in this study. Our study also included individuals who were at an increased risk for DR-TB and patients who had defaulted on their ATT. Patients with poor adherence, such as irregular dosing or premature cessation of therapy, and newly diagnosed TB patients who had recent close contact with known DR-TB cases were also included (Singh et al., 2022a).

On the other hand, patients were excluded if they tested smear-negative through fluorescent microscopy, as microbiological confirmation was essential for inclusion. Samples with invalid LPA results were also excluded because they could not provide reliable resistance data.

### 2.3 Sample processing

The samples were processed in a Class III biosafety cabinet within a Biosafety Level 3 (BSL-3) laboratory using the standard NALC-NaOH method. Sterile tubes and autoclaved reagents were used. For decontamination, we used 3–5 mL of 1.5% NaOH solution and allowed it to stand for 20 min, followed by neutralization with PBS (Singh et al., 2025). The samples were then centrifuged, and the pellet was resuspended in PBS. An aliquot of 1.8 mL was stored at 4 °C. A high-quality smear was prepared for fluorescence microscopy.

### 2.4 Microscopy

After air-drying the smear, auramine dye was applied to stain *M. tuberculosis.* Slides were decolorized with acid-alcohol and counterstained with potassium permanganate for contrast. Stained slides were air-dried and examined under a fluorescence microscope.

### 2.5 Line probe assay for DR-TB detection

For DNA extraction, 500 μL of decontaminated and processed sample was centrifuged. The resulting pellet was then resuspended in 100 μL of lysis buffer (A-LYS) and allowed to sit at 95 °C. After cooling, 100 μL of neutralization buffer (A-NB) was added, and the supernatant was utilized for polymerase chain reaction (PCR) amplification following the guidelines provided by the MTBDR*plus* manufacturer (MTBDR Manual, 2015).

The supernatant containing DNA was stored for PCR. After this, we performed polymerase chain reaction (PCR), during which DNA was amplified using AM-A and AM-B reagents in a thermal cycler (Singh et al., 2022a). PCR cycles included denaturation (DEN; 95 °C), annealing (65 °C), and extension (70 °C). Finally, hybridization (HYB) was performed in a 45 °C water bath using DEN and HYB buffers. DNA was hybridized on strips, washed (STR, RIN), and then incubated with conjugate (CON) and substrate (SUB) solutions. Color development in form of bands indicated the presence or absence of resistance genes.

### 2.6 Result interpretation

After hybridization, the strip was removed and dried, and the results were interpreted. Strips were pasted on reporting paper and stored away from light. We used an evaluation sheet and pasted the developed strips in the designated fields by aligning the bands conjugate control (CC) and amplification control (AC) with the respective lines on the sheet. Each strip of LPA had 27 reaction bands, including 7 controls. The presence of bands conferring mutational genes of drug-specific test lines or the absence of wild-type gene indicated resistance for *rpo*B, *kat*G, and *inh*A gene.

### 2.7 Statistical analysis

Microsoft Excel was used for data collection, and SPSS 15.0 (SPSS, Inc., Chicago, IL, United States) was used to evaluate study results. Mean ± standard deviation was used for indicating qualitative values, whereas numbers and percentages were used to represent quantitative factors. Categorical variables were compared using chi-squared test where appropriate, while continuous variables such as age and BMI were analyzed using Student’s *t*-test after testing for normality. A *p*-value of < 0.05 was considered statistically significant. The results were also expressed with 95% confidence interval to provide precision of estimates.

## 3 Results

**Figure 1:**
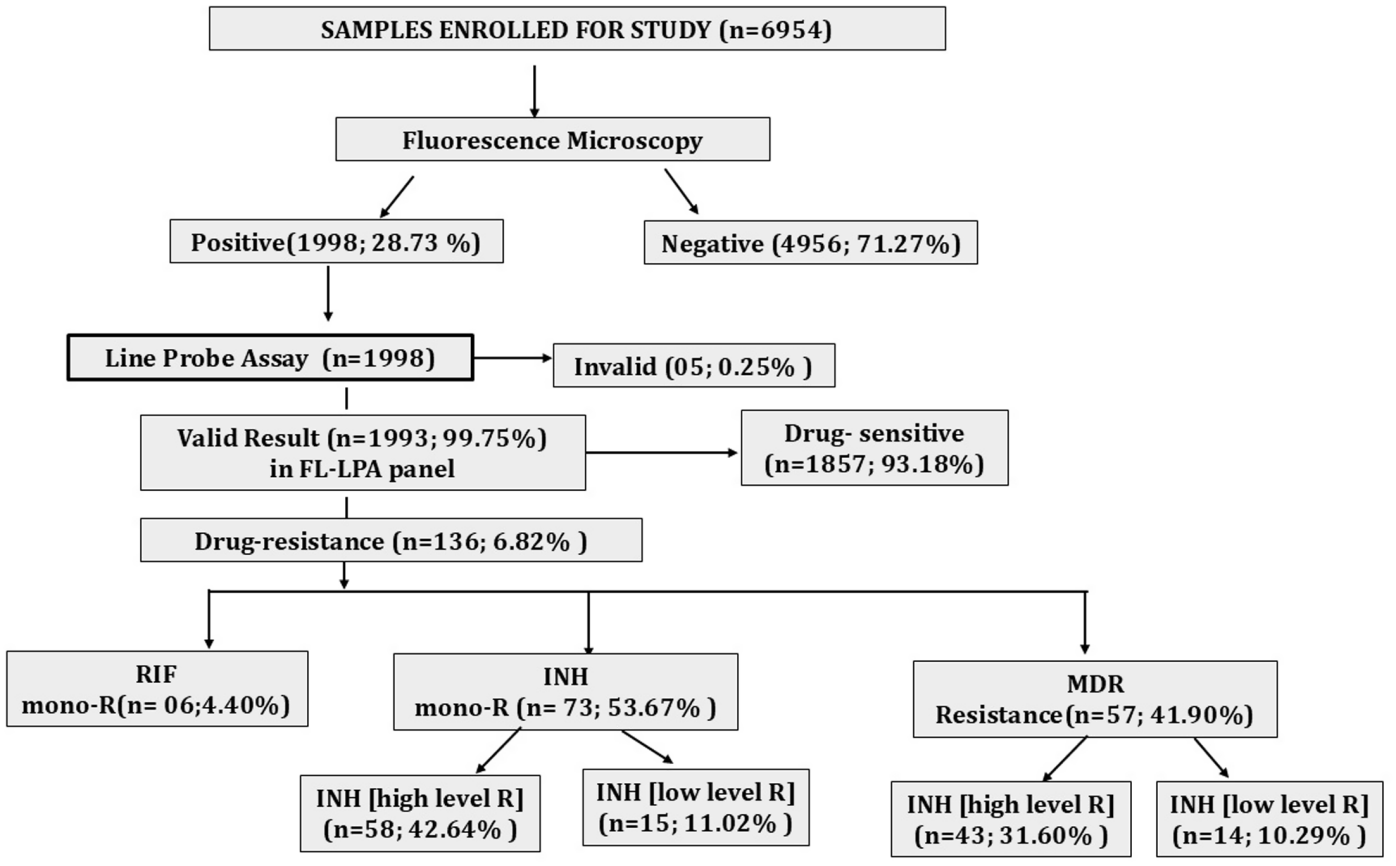
Diagnostic cascade showing total samples tested by fluorescence microscopy, percentage positivity, and proportion of DR- TB detected. mono-R, mono resistance; RIF, rifampicin; INH, isoniazid; MDR, multidrug resistance.

We included a total of 6,954 clinical samples and tested them using fluorescence microscopy for the confirmation of TB. A total of 1,998 samples (28.73%) were found to be smear-positive, whereas the remaining 4,956 samples (71.27%) were smear-negative. Furthermore, molecular testing of smear-positive samples using the GenoType MTBDR*plus* assay revealed 136 cases of DR-TB (6.82%). Among these 136 cases, 6 cases were RIF mono-resistant, 73 cases were INH mono-resistant, and 57 cases were MDR-TB (Figure 1).

### 3.1 Demographic and clinical characteristics

We compared the demographic and clinical characteristics of DR-TB cases with drug-sensitive tuberculosis (DS-TB) cases, as mentioned in Table 1. However, several other factors have been explored for their impact on these outcomes, such as age, gender, treatment history, and TB source. The average age ± SD of patients with DR-TB was quite less as compared to DS-TB patients (36.54 ± 16.27 vs. 38.67 ± 18.10 yrs., *p* = 0.70) which was statistically insignificant. In terms of gender-associated average age, the average age for male DR-TB patients was 38.67 ± 13.35, and for male DS-TB patients it was 42.43 ± 17.85 years (*p*-value = 0.192), which was statistically non significant. The mean age of female DR-TB patients with resistance to RIF was 27.30 ± 13.11 years, whereas it was 32.46 ± 16.83 years in the female DS-TB patients (*p*-value = 0.146), indicating no significant difference in the ages of women in both DS and DR-TB population. In DR-TB resistance cases, women were significantly younger than males. In gender distribution, 90 (66.17%) men had DR-TB, while 1,135 (61.62%) had DS-TB. Among women, 46 (33.82%) had DR-TB and 707 (38.11%) had DS-TB. The *p*-value of gender distribution was 0.47 between the two groups, indicating a statistically non-significant difference between the ratio of men and women among DR-TB and DS-TB cases. However, the prevalence of DR-TB was higher in men than in women.

**Table 1:** Demographic and clinical comparison between DR-TB and DS-TB patients.

Demography		DR-TB cases (*n* = 136)	DS-TB cases (*n* = 1,857)	*p*-value
Age (mean ± SD*) in years		36.54 ± 16.27	38.67 ± 18.10	0.70
Distribution				
Age distribution (mean ± SD*)	Men	38.67 ± 13.35^a^	42.43 ± 17.85	0.192
	Women	27.30 ± 13.11^a^	32.46 ± 16.83	0.146
Gender-wise distribution	Men	90 (66.17%)	1,135 (61.62%)	0.475
	Women	46 (33.82%)	707 (38.11%)	0.673
Treatment history				
New cases		60 (44.11%)	1,834 (99.56%)	–
Previously treated cases		76 (55.88%)	11 (0.44%)	≤0.001
Source of TB infection				
History of TB in family		02 (1.47%)	05 (0.26%)	–
History of TB in relatives		03 (2.20%)	01 (0.01)	–
History of TB in neighbor		09 (6.61%)	09 (0.48%)	–
History of TB at workplace		16 (11.76%)	14 (0.76%)	≤0.001
Unknown source		106 (77.94%)	1813 (97.67%)	–
District-wise distribution				
Gorakhpur		48 (35.30%)	526 (28.32%)	–
Basti		03 (2.2%)	24 (1.29%)	–
Deoria		33 (24.30%)	846 (45.56%)	≤0.001
Sant Kabirnagar		06 (4.4%)	108 (5.81%)	–
Siddharthnagar		23 (16.90%)	37 (1.99%)	–
Kushinagar		19 (14.00%)	281 (15.13%)	–
Maharajganj		04 (2.9%)	39 (2.10%)	–

DS-TB, drug-sensitive tuberculosis; DR-TB, drug-resistant tuberculosis; SD, standard deviation.

Treatment history is another important factor analyzed in this research. Among DR-TB cases, 60 patients (44.11%) were newly diagnosed, and 76 (55.88%) had been treated previously. In the case of DS-TB, 1,834 (99.56%) were newly diagnosed and only 11 (0.44%) were previously treated. Prior treatment cases were significantly higher in the DR-TB group compared to DS-TB cases (76 [55.58%] vs. 11 [0.44%], *p* ≤ 0.001), as shown in Table 1. The increased proportion of previously treated cases in the DR-TB group indicates unsuccessful or inadequate treatment, a hallmark of resistant TB.

The source of infection was also compared between the two groups. In cases of DR-TB, 1.47, 2.20, 6.61, and 11.76% patients had family, a relative, a neighbor, and workplace as the source of infection, respectively. Compared to this, in case of DS-TB, 0.26% patients had family, 0.02% had a relative, 0.48% had a neighbor, and 0.76% had workplace as the source of infection, respectively. The source of infection from workplace for DR-TB cases was significantly higher than DS-TB cases (16 [11.76%] vs. 14 [0.76%], *p* ≤ 0.001). Unknown source of infection was the most prominent, and 77.94% of DR-TB cases and 97.63% of DS-TB cases were classified under this category.

Out of a total of 2,316 TB cases, 136 (6.82%) were DR-TB cases, while 1,857 (93.17%) were DS-TB. Gorakhpur reported 48 (35.30%) cases among total DR-TB cases and 526 (28.32%) were DS-TB, as shown in Table 1. Siddharthnagar had 23 (16.90%) DR-TB cases compared to 37 (1.99%) DS-TB. Basti had 3 (10.34%) DR-TB cases and 24 (1.29%) DS-TB. Deoria reported 18 (1.87%) DR-TB cases and 846 (45.56%) DS-TB. In Kushinagar, 12 (3.21%) cases were of DR-TB, while 281 (15.13%) cases were DS-TB. Sant Kabirnagar reported 6 (4.40%) DR-TB cases and 108 (5.81%) DS-TB. In Maharajganj, only 4 (2.9%) cases were DR-TB, while 39 (1.90%) were DS-TB.

### 3.2 Genetic mutational patterns

**Figure 2:**
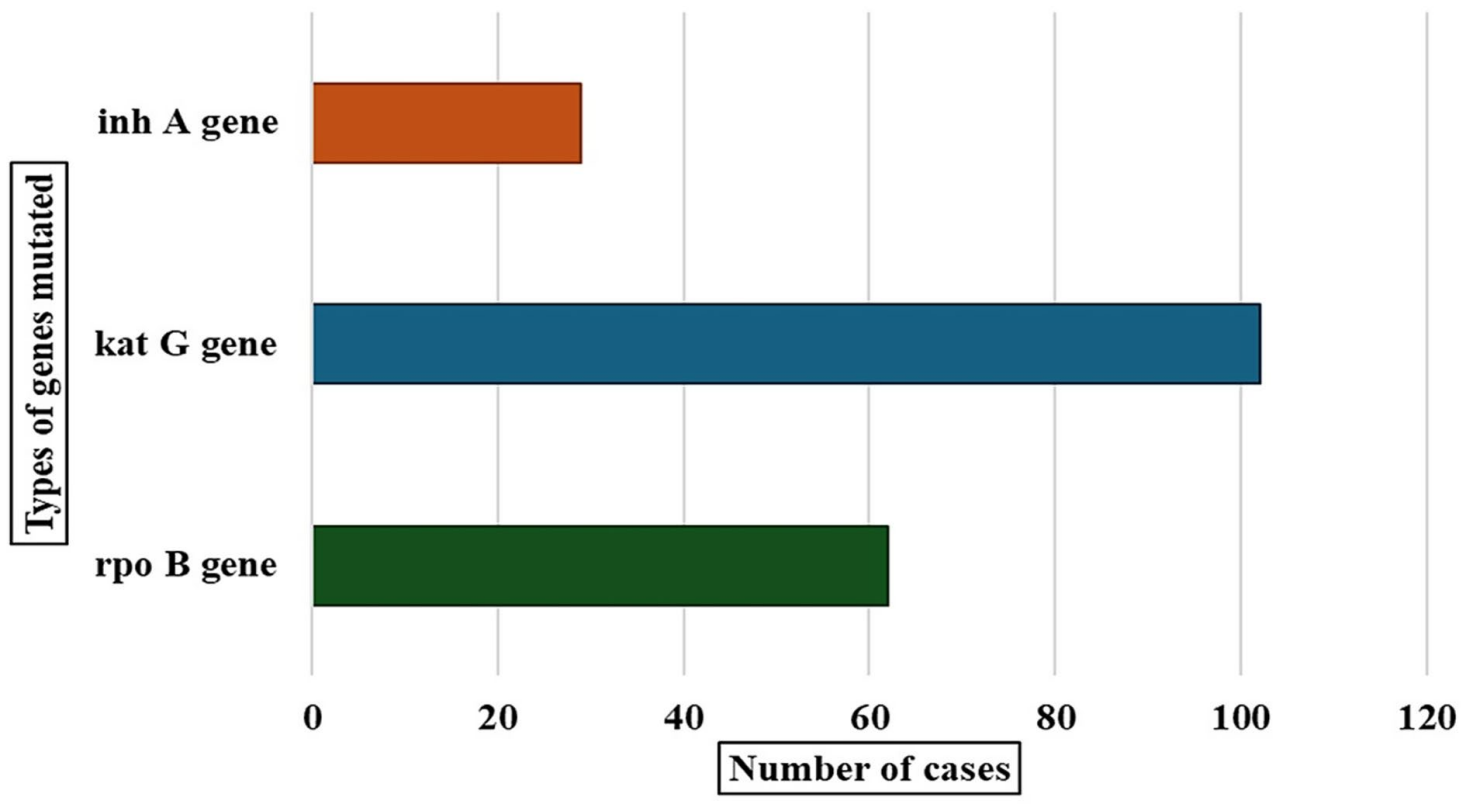
Distribution of different gene mutations observed among DR-TB cases.

Our study helped identify gene mutations in drug-resistant tuberculosis cases, and we detected mutations in three genes: *rpo*B, *kat*G, and *inh*A (Figure 2). The *kat*G gene showed the highest number of mutations in 101 cases. The *rpo*B gene had mutations in 63 cases, and the *inh*A gene showed mutations in 29 cases.

We also examined gene mutations related to *rpo*B, *kat*G, and *inh*A genes. Among 63 cases analyzed for *rpo*B mutations, the S531L (*rpo*B MUT3) mutation was the most prevalent, observed in 51 cases, accounting for 82.25% of the total cases. The H526Y (*rpo*B MUT2A) mutation was found in 11 cases, accounting for 17.75%. Both mutations were located at codon regions 530–533 and 526–529, respectively (Table 2). For the *kat*G gene, 130 cases were evaluated, and the S315T1 (*kat*G MUT1) mutation was detected in 83 cases, representing 63.35%. Additionally, the S315T2 variant of the same codon was present in 18 cases, contributing to 14.50% of the total. In the *inh*A gene, also analyzed in 130 cases, the C-15 T (*inh*A MUT1) mutation was observed in 29 cases, accounting for 22.13%.

**Table 2:** Gene-specific mutation profiles along with corresponding codons in DR-TB isolates.

Resistance gene	Mutation probe	Codon analysis	No. of cases (%)
*rpo*B	*rpoB* MUT2A (H526Y)	526–529	11/63 (17.75%)
	*rpoB* MUT3 (S531L)	530–533	51/63 (82.25%)
*kat*G	*katG* MUT1 (S315T1)	315	83/130 (63.35%)
	*katG* MUT1 (S315T2)	315	18/130 (14.50%)
*inh*A	*inhA* MUT1 (C-15T)	−15	29/130 (22.13%)

### 3.3 Treatment outcomes and clinical profiling of DR-TB cases

Among the 136 DR-TB patients analyzed, 94 (69.1%) were cured, 12 (8.8%) remained on ongoing treatment, 24 (17.6%) were lost to follow-up, and 6 (4.4%) died. The mean age of patients was 37.6 ± 16.3 years in the cured group and 44.8 ± 27.1 years in those who died. Most patients had a BMI < 20, and comorbidities such as diabetes (15/136) and anemia (7/136) were observed more frequently in patients with unfavorable outcomes. Symptomatic profiling showed that cough, fever, weight loss, and reduced appetite were the most common features across all groups.

## 4 Discussion

The global burden of DR-TB represents a major public health challenge, particularly in areas with high TB prevalence. Meanwhile, a study showed that global prevalence of DR-TB among pediatric patients was 13.59%, with MDR-TB constituting 3.72%, which is higher than our study that showed 6.82% of DR-TB (Song et al., 2021). This highlights the worldwide challenge of DR-TB, especially in regions with poor healthcare systems. Our study also demonstrated a high validity rate of LPA, consistent with earlier studies that reported high sensitivity and specificity for detecting RIF and INH resistance (Nathavitharana et al., 2017).

Under routine NTEP settings, the current standard of care for DR-TB includes the use of Cartridge-Based Nucleic Acid Amplification Test for rapid screening, followed by LPA and culture-based Drug Susceptibility Testing for confirmation (Singh et al., 2022b). Our findings on mutation profiles at the district level provided important molecular evidence that can complement these diagnostics and support early, individualized treatment decisions, thereby informing policy for decentralized TB control. LPA has proven to be a recognized molecular method, well-known for its high sensitivity and specificity in detection of RIF- and INH-related gene mutations (Kebede et al., 2017; Aricha et al., 2019). However, it has some limitations such as a limited sensitivity in smear-negative cases, the need for specialized laboratory infrastructure, and the inability to detect rare resistance mutations outside of the targeted gene regions (NTEP, 2021).

Our study observed age distribution with a mean age of DR-TB patients as 36.54 ± 16.27 yrs., with male patients averaging 38.67 ± 13.35 years and females 27.30 ± 13.11 years. In contrast, a meta-analysis showed a mean age of 37.5 years for DR-TB patients (Atif et al., 2022). Another meta-analysis study found that DR-TB rates varied by age group and exhibited approximately 4.0% of MDR-TB (Song et al., 2021). This variation suggests age-related patterns in the prevalence of DR-TB. The TB Report 2024 of India also highlighted a similar trend, where younger individuals were disproportionately affected by MDR-TB, particularly in regions with gaps in effective TB control measures (India TB Report, 2024). The regular age of DR-TB patients in India was reported to be between 30 and 40 years, highlighting the higher risk among younger individuals. We studied the prevalence of DR-TB in seven districts and found that the resistance pattern of drugs against TB in these areas was not similar. The districts Deoria and Gorakhpur had the most resistant TB cases, which could indicate the presence of a localized DR-TB epidemic requiring focused intervention. These differences in resistance patterns across geographical gradients might be due to the way TB control programs are implemented, the socioeconomic structure of the area, and the extent of health facilities.

Our study showed that men had a higher predisposition to drug resistance compared to women and revealed a significant male predominance in DR-TB cases, accounting for 64.53% of patients. This was similar to other global observations where men were typically more impacted by DR-TB than women. A significant research found that male proportion was higher at 53.1% among MDR-TB patients (Atif et al., 2022). The Global TB Report by WHO also supported this finding and mentioned that men account for a larger proportion of MDR-TB cases globally (WHO, 2024). Another remarkable study indicated a lower rate of MDR-TB in females compared to males in both economically developed and developing countries, aligning with the male predominance seen in our studies (Song et al., 2021). Approximately 60% of all MDR-TB cases worldwide were reported among men, reflecting the global demographic trend of higher male susceptibility to DR-TB. The TB Report by India also reported male predominance, with 70% of MDR-TB cases in men, underscoring the gender-specific risk factors associated with DR-TB in high-burden countries like India (India TB Report, 2024). In our study, 1993 (28.56%) were confirmed positive TB cases among 6954 suspected cases. In contrast, an impactful study had observed a positivity rate of only 18% among 1,303 suspected cases (Javed et al., 2018). The higher positivity rate detected in our study might be possible due to variances in patient selection criteria, diagnostic protocols, and/or regional TB burden.

A multicenter study had observed that the prevalence of previous TB treatment history in DR-TB cases was also notably high (Nathavitharana et al., 2017). Another significant study found that those who had a TB treatment history were at increased risk of acquiring MDR-TB (Mor et al., 2014). Both studies had relevance to our results, as we also showed a higher risk of DR-TB in previously treated group. Another study emphasized that transmission-driven resistance was more prominent in high-burden areas, often affecting younger populations (Farhat et al., 2024). However, they also acknowledged that prior an incomplete treatment of TB was the main cause of the resistance development. In regions with limited access to appropriate treatment, incomplete prior treatment history was an important contributor to high incidence of MDR-TB. This observation showed concordance with our findings, where a large portion of patients with DR-TB had been treated earlier. A study showed the predominant genetic lineage among resistant strains was Euro-American, comprising 13 (76.5%) of drug-resistant isolates, indicating regional variation (Gashaw et al., 2021).

Our study revolved around RIF resistance due to mutations in the *rpo*B gene, of which *rpo*B MUT3 (S531L) was the most prevalent, found in 51 (82.25%) cases, followed by *rpo*B MUT2A (H526Y) type mutation detected in 11 (17.75%) cases. Another study showed S531L type mutation in 63.8% cases and H526Y type mutation in 2.9% cases, which is lower than our findings (Maharjan et al., 2017). Both studies observed similar genetic mutations in RIF-resistant strains, suggesting that the S531L mutation is widespread and remains the most prevalent mutation despite differences in geographical locations. A remarkable study found *rpo*B MUT3 in 6/7 (85.72%) RIF-resistant cases and *rpo*B MUT2A in 1/7 (14.28%) cases (Giri, 2025). A study also noted S531L type of *rpo*B mutations in 1,483/1,970 (75.3%) cases (Jain et al., 2016). Both studies had concordance with our study. Co-resistance refers to simultaneous resistance to more than one anti-tubercular drug, such as rifampicin and isoniazid. Another finding showed S531L type of mutation was found in 61.4% cases, which is significantly lower than our findings (Vashistha et al., 2017). These disparities further reinforce the significance of these mutations across different geographical locations. These findings also reflect both consistent patterns and regional variations, which are influenced by multiple factors like healthcare facilities, prior treatment history, and local TB control strategies.

Another impactful study found that RIF resistance is linked to *rpo*B mutations, particularly S531L (Rashid et al., 2020). These findings were in concordance with our study, which also identified *rpo*BS531L as the predominant mutation responsible for RIF resistance. These findings are consistent with a study in which the *rpo*B MUT3 (S531L) mutation was the most common, reported in 54.9%, and *rpo*B MUT2A appeared in 11.8% of MDR-TB isolates (Liu et al., 2017), and these observations were similar to our research. These results suggest that the *rpo*BS531L mutation is a key marker for RIF resistance. Furthermore, there is one more study that similarly highlighted the strong association between mutations in the *rpo*B gene and RIF resistance (Dubey et al., 2017). It also showed frequent presence of S531L mutation in RIF-resistant strains. The consistency of the *rpo*B MUT3 (S531L) mutation in various studies underscores its importance as a diagnostic marker for RIF. The diagnostic cascade included the total number of samples tested by fluorescence microscopy, followed by assessment of drug resistance across different populations. The identification of this mutation using molecular diagnostic methods, such as GenoType MTBDR*plus* assay, can significantly expedite the diagnosis and management of MDR-TB. Genetic analysis revealed RIF-resistant mutations in the *rpo*B gene and, combined with other gene mutations in *rpo*B, *kat*G, and *inh*A, were significantly linked to fluoroquinolone resistance, indicating a complex genetic pattern of multidrug resistance. Another study highlighted the challenges in Ukraine due to limited access to advanced molecular testing outside specialized centers, contributing to delays in appropriate treatment (Dudnyk et al., 2024). Gender and age distributions were more detailed in the study by Dudnyk et al., whereas Shanu et al. focused more on diagnostic performance. Our molecular profile corresponds precisely with a study that identified the S531L mutation as the most frequent globally, often detected by commercial molecular assays (Farhat et al., 2024).

A study showed INH resistance due to *kat*G mutations in 8 (66.7%) cases, which was mainly at codon 315, and *inh*A mutations found in fewer isolates at positions −15/−16 (Gashaw et al., 2021), which is nearly similar to our study. Our findings showed *kat*G gene mutation in 101/130 (78.46%) INH-resistant cases with S3151T1 in 83 (63.30%) cases and S315T2 in 18 (14.50%). Our findings are quite similar to a study that emphasized the importance of early detection of INH resistance, particularly high-level resistance mediated by *kat*G mutations (Charoenpak et al., 2020). This study showed high prevalence of *kat*G mutations, 79.54%, in DR-TB cases (Charoenpak et al., 2020). Our study also showed 22.13% of INH-resistant cases had mutations in *inh*A gene. Our findings were further supported by a multicentric study, which also identified the *inh*A C-15 T mutation as a marker for low-level resistance (Dean et al., 2020). Another study showed 68.05% *kat*GS315T1 type of mutation, which is similar to our study (Kebede et al., 2017). Our study is also consistent with a remarkable study that observed 93.6% *kat*GS315T1 and 1.8% *kat*GS315T2 types of mutations, which is significantly higher than our study (Jain et al., 2016).

An impactful study also supported the role of the *kat*G gene in INH resistance. It showed that INH resistance was most commonly linked with mutations in the *kat*G gene, particularly the S315T1 mutation (Dubey et al., 2017). Another study found 79.7% of INH-resistant isolates carrying mutations in the *kat*G gene, specifically S315T, which is a hallmark of high-level INH resistance (Maharjan et al., 2017). Our findings align with this study, as we observed *kat*G mutations in 71% of INH-resistant cases, confirming the global dominance of S315T in high-level resistance. The slight difference in prevalence could be due to the regional variability in treatment practices, as Nepal has been facing challenges related to incomplete TB treatment and non-adherence in certain areas, leading to higher mutation rates. Our results were in concordance with the findings of a research that reported *kat*G mutations as the primary cause of INH resistance in MDR-TB isolates, with *kat*G MUT1 (S315T1) present in 66.7% of such strains (Liu et al., 2017). It also observed the *inh*A mutation in 18.1% of MDR-TB cases, which is similar to our findings.

This further reinforced the notion that *kat*G mutations, particularly the S315T1 variant, were related to high-level INH resistance. This aligns closely with our study’s observations and further strengthens the genetic association between *kat*G mutations and high-level INH resistance across different settings. The native variation in low-level resistance may reflect the local prevalence of specific strains and the varying drug pressure exerted by national TB control programs. The findings from South Africa showed that 80.6% of INH-resistant patients were high-level resistant and frequency of *kat*G mutations were in concordance with our data. The prevalence of *kat*GS315T mutations in retreatment cases in our cohort was consistent with these studies, indicating that prior treatment plays a serious role in the high-level resistance development (Bokop et al., 2023). Similarly, a global analysis of 211,753 TB patients across 156 countries revealed that *kat*G mutations were present in 78.6% of INH-resistant isolates. Our findings corroborated these global trends, further supporting the pivotal role of *kat*G mutations in mediating high-level resistance.

A notable difference lies in the presence of additional mutations. We also observed mutations in the *inh*A gene at the promoter region of C-15T, where 20.85% of the low-level INH-resistant cases. This pattern mirrors findings in which the *inh*A mutation was linked to low-level resistance and was present in 15 isolates (Charoenpak et al., 2020). The correlation between *inh*A mutations and lower resistance levels was also reflected in the study, which observed that the *inh*A C-15 T mutation contributed to low-level resistance in 6.8% of isolates (Dean et al., 2020). The variations in drug resistance patterns across regions could be responsible for numerous issues, including the healthcare infrastructure, regional & national TB control strategies, and treatment history of patients. For example, regions lacking adequate quality healthcare, like Namibia, tend to have a higher frequency of DR-TB due to incomplete anti-TB treatment and non-adherence to drug regimens. Regional variations in the frequency of specific strains and the history of TB treatment showed an important role in shaping the mutational patterns observed in INH-resistant cases. These regional differences highlighted the need for localized data to design effective public health strategies and personalized treatment plans for DR-TB. Another study highlighted the dual necessity of *kat*G and *inh*A testing for comprehensive detection, an approach adopted in our methodology (Farhat et al., 2024). A study underlined the importance of simultaneous detection of these two mutations to prevent underdiagnosis of INH mono-resistance, validating our dual-target strategy (Nathavitharana et al., 2017).

Our study of 172 DR-TB patients showed a treatment success rate of 69%, with 16% lost to follow-up, 11% having incomplete treatment, and 3.5% mortality, primarily among older patients with low BMI. Key symptoms such as fever (98.3%), reduced appetite (95.8%), and weight loss (95.8%) were highly prevalent in cured patients, while smoking (7.4%) and alcohol use (11.1%) were more common in those with poor outcomes. Our findings are consistent with a significant study that reported a treatment success rate of 71.8% with a male predominance. The study also highlighted that a low BMI (<18.5) serves as a predictor of poor outcomes (Baluku et al., 2021). However, unlike our cohort, their study demonstrated a higher mortality rate, showing some discordance with our results. Similarly, another study reported a 65% treatment success rate in India, which is comparable to our findings (Bhatt et al., 2019). In addition, a study from Ethiopia documented a treatment success rate above 65% and a loss to follow-up rate of 16.8%, closely aligning with our cohort (Molie et al., 2019). These comparisons reinforce that while treatment success rates are improving across different regions, challenges such as loss to follow-up, older age, and comorbidities continue to limit outcomes, underscoring the need for comprehensive patient support systems.

## 5 Future directions

Future studies should extend beyond first-line drug resistance to include in-depth analysis of second-line anti-tubercular drugs, particularly fluoroquinolones and group A drugs, which are increasingly associated with XDR-TB. Whole-genome sequencing and advanced molecular approaches could further elucidate emerging resistance mechanisms and transmission dynamics at the community level. These approaches are critical to inform individualized treatment regimens and strengthen TB control strategies under NTEP.

## 6 Conclusion

This study offers significant insights into the molecular epidemiology of drug-resistant tuberculosis (DR-TB) in Eastern Uttar Pradesh, India. The findings highlight a high prevalence of specific mutations—particularly *rpo*B MUT3 (S531L) for rifampicin resistance and *kat*G MUT1 (S315T1) for high-level isoniazid resistance—underlining the critical role of molecular diagnostics such as line probe assay (LPA) in rapid and accurate detection. The demographic analysis showed a higher incidence of DR-TB among men and previously treated patients, with workplace exposure emerging as a notable risk factor. District-wise variation in resistance patterns suggests localized transmission dynamics and treatment practices influencing the evolution of resistance. The detection of *inh*A C-15 T mutations further stresses the importance of distinguishing between low- and high-level isoniazid resistance to guide appropriate treatment. Incorporating LPA into routine TB diagnostic workflows enables early detection, reduces diagnostic delay, and aids in personalized treatment regimens. Our findings support the need for region-specific TB control strategies, enhanced surveillance, and strict adherence to treatment protocols to curb the spread of DR-TB. Continued molecular surveillance and integration of genetic profiling into national TB programs are crucial for achieving TB elimination goals. Future research should focus on second-line drug resistance patterns and the evolution of new mutations to strengthen the public health response against DR-TB.

## 7 Limitation of study

This study was conducted at a single tertiary care teaching hospital and included 6,954 samples from referral patients, which may not be representative of the general population. Therefore, multicentric studies involving larger and more diverse populations are needed to validate and generalize these findings. The absence of follow-up data limited the assessment of clinical outcomes in relation to the detected genetic mutations.

## Data Availability

The original contributions presented in the study are included in the article/supplementary material, further inquiries can be directed to the corresponding author.
